# Supplementary material for: All-angle Negative Reflection with An Ultrathin Acoustic Gradient Metasurface: Floquet-Bloch Modes Perspective and Experimental Verification
Source: Sci Rep. 2017 Oct 23;7:13852. doi: 10.1038/s41598-017-14387-9 (PMC5653785; doi:10.1038/s41598-017-14387-9)
Supplement: Supplementary file 1 — Supplementary material [file 41598_2017_14387_MOESM1_ESM.pdf]

Supplementary material for

"All-angle Negative Reflection with an Ultrathin Acoustic Gradient Metasurface: Floquet-Bloch Modes Perspective and Experimental Verification"

Bingyi Liu, Jiajun Zhao, Xiaodong Xu, Wenyu Zhao and Yongyuan Jiang

In this supplementary part, we offer the detailed information about the mode expansion theory for acoustic gradient metasurface.

The incident acoustic plane wave illuminating at angle θ_i can be written as

$$\begin{cases} p^{in}(\vec{r},t) = \exp\left[i\left(k_x^{in}x - k_z^{in}z - \omega t\right)\right] \\ \vec{v}^{in}(\vec{r},t) = \dfrac{1}{Z_0}\exp\left[i\left(k_x^{in}x - k_z^{in}z - \omega t\right)\right]\left[\dfrac{k_x^{in}}{k_0}\hat{x} - \dfrac{k_z^{in}}{k_0}\hat{z}\right] \end{cases}, \tag{S-1}$$

where $k_0 = \omega/c_0$ is the amplitude of wave vector in air, ω is the angular frequency, c_0 is the sound speed of air and $Z_0 = \rho_0 c_0$ is the acoustic impedance of air, moreover, $k_x^{in} = k_0 \sin\theta_i$ and $k_z^{in} = \left[k_0^2 - \left(k_x^{in}\right)^2\right]^{1/2}$. The total acoustic field in region I in Fig. 2 can be written in the form of Rayleigh expansions

$$\begin{cases} p^I = p^{in} + \sum\limits_n R_n p_n^r \\ \vec{v}^I = \vec{v}^{in} + \sum\limits_n R_n \vec{v}_n^r \end{cases}, \tag{S-2}$$

and

$$\begin{cases} p_n^r = \exp\left[i\left(k_x^{r,n}x + k_z^{r,n}z - \omega t\right)\right] \\ \vec{v}_n^r = \dfrac{1}{Z_0}\exp\left[i\left(k_x^{r,n}x + k_z^{r,n}z - \omega t\right)\right]\left[\dfrac{\left(k_x^{in} + nG\right)}{k_0}\hat{x} + \dfrac{k_z^{r,n}}{k_0}\hat{z}\right] \end{cases}, \tag{S-3}$$

where $\left(k_x^{r,n}\right)^2 + \left(k_z^{r,n}\right)^2 = k_0^2$ and $k_x^{r,n} = k_x^{in} + nG\left(n = 0, \pm 1, \pm 2, \ldots\right)$. L is the length of one supercell period, $G = 2\pi/L$ refers to the amplitude of the supercell reciprocal lattice vector and R_n stands for the scattering coefficient corresponding to the n-th order diffraction. It is obvious that equation (S-2) and (S-3) exactly treats the acoustic gradient metasurface as a generalized acoustic grating or phononic crystal that the

influence of nonlocal effect originates from system periodicity can not be neglected. Inside the region II, the density and the bulk modulus of the metasurface slab are functions of x positions, the corresponding pressure and the velocity field can be solved by relations $\vec{\rho}\frac{\partial \vec{v}}{\partial t}=-\nabla p$ and $\frac{\partial p}{\partial t}=-\nabla\cdot\vec{B}\vec{v}$, which deduce

$$\begin{cases}\dfrac{\partial p^{II}}{\partial x}=ik_0Z_0\rho_x\left(x\right)v_x^{II}\\ \dfrac{\partial p^{II}}{\partial z}=ik_0Z_0\rho_z v_z^{II}\\ \dfrac{\partial\left[B_0B_x\left(x\right)v_x^{II}\right]}{\partial x}+B_0B_z\dfrac{\partial v_z^{II}}{\partial z}=i\omega p^{II}\end{cases}, \tag{S-4}$$

and thus the eigen modes of the sound waves propagating inside the metasurface slab can be solved by the following differential equation

$$\frac{B_z}{\rho_z}\frac{\partial^2 p^{II}}{\partial z^2}+\frac{\partial}{\partial x}\left(\frac{B_x}{\rho_x}\frac{\partial p^{II}}{\partial x}\right)+\frac{\rho_0}{B_0}\omega^2 p^{II}=0. \tag{S-5}$$

which is exactly the equation (3) proposed in the main article. According to the Floquet theorem, we can rewrite the acoustic pressure and the z-component of the velocity field inside the metasurface slab as

$$\begin{cases}p_{\pm}^{II}\left(q_z,x,z\right)=G\left(q_z,x\right)\mathrm{e}^{\mp iq_z z}=\left[h_1g^{+}\left(q_z,x\right)+h_2g^{-}\left(q_z,x\right)\right]\mathrm{e}^{\mp iq_z z}\\ v_{z,\pm}^{II}\left(q_z,x,z\right)=\dfrac{\mp q_z}{k_0Z_0\rho_z}G\left(q_z,x\right)\mathrm{e}^{\mp iq_z z}\end{cases}, \tag{S-6}$$

It should be noted that here we generalize the field components inside the metasurface slab by only considering the left-going and right-going modes. Then equation (S-5) can be written as

$$\frac{B_x}{\rho_x}\frac{\partial^2 G\left(q_z,x\right)}{\partial x^2}+\frac{\partial}{\partial x}\left(\frac{B_x}{\rho_x}\right)\frac{\partial G\left(q_z,x\right)}{\partial x}+\left(\frac{\rho_0}{B_0}\omega^2-\frac{B_z}{\rho_z}q_z^2\right)G\left(q_z,x\right)=0, \tag{S-7}$$

For the real realization of the gradient metasurface depicted in Figure 2(b), we need to discretize equation (S-7) for further analysis. Discretizing equation (S-7) we can obtain M linear equations

$$\sum_{m'}H_{mm'}G_{m'}=q_z^2G_m, \tag{S-8}$$

where $H_{mm'}$ is a $M\times M$ matrix, its element is given by

$$H_{mm'} = \left(\frac{\rho_0}{B_0}\omega^2 - 2\gamma\frac{B_{x,m}}{\rho_{x,m}}\right)\delta_{mm'} + \gamma\left[\frac{B_{x,m}}{\rho_{x,m}} + \left(\frac{B_{x,m-1}}{\rho_{x,m-1}} - \frac{B_{x,m+1}}{\rho_{x,m+1}}\right)\right]\delta_{m,m'-1} + \gamma\left[\frac{B_{x,m}}{\rho_{x,m}} + \left(\frac{B_{x,m+1}}{\rho_{x,m+1}} - \frac{B_{x,m-1}}{\rho_{x,m-1}}\right)\right]\delta_{m,m'+1}, \qquad \text{(S-9)}$$

and $\gamma = (2l)^{-2}$. It should be noted that there exists two off-diagonal matrix elements $H_{1,M}$ and $H_{M,1}$ corresponding to the boundary indexes at x=0 and x=L. Solving equation (S-8), M eigenvalues $q_{z,j}^2$ can be obtained. These plane wave solutions encapsulated by discretized unit elements function similarly as the induced currents of the electromagnetic metasurface.

Using the Floquet-Bloch boundary condition

$$G(q_z, x+L) = \exp(ik_0 L\sin\theta_i)G(q_z, x), \qquad \text{(S-10)}$$

therefore, two off diagonal matrix elements turn out to be

$$H_{1,M} = \gamma\left[1 + \frac{\rho_{x,1}}{B_{x,1}}\left(\frac{B_{x,2}}{\rho_{x,2}} - \frac{B_{x,M}}{\rho_{x,M}}\right)\right]\exp(ik_0\sin\theta_i L)$$
$$H_{M,1} = \gamma\left[1 + \frac{\rho_{x,M}}{B_{x,M}}\left(\frac{B_{x,M-1}}{\rho_{x,M-1}} - \frac{B_{x,1}}{\rho_{x,1}}\right)\right]\exp(-ik_0\sin\theta_i L), \qquad \text{(S-11)}$$

thus we can solve the allowed waveguide modes stimulated inside the metasurface slab. Now, the total acoustic pressure field in the metasurface slab can be written as

$$p^{II}(x,z) = \sum_j S^+(q_{z,j})\,p_+^{II}(q_{z,j},x,z) + S^-(q_{z,j})\,p_-^{II}(q_{z,j},x,z)$$
$$= \sum_j \left[S^+(q_{z,j})\mathrm{e}^{-iq_{z,j}z} + S^-(q_{z,j})\mathrm{e}^{iq_{z,j}z}\right]G(q_{z,j},x)$$
$$v_z^{II}(x,z) = \sum_j S^+(q_{z,j})\,v_{z,+}^{II}(q_{z,j},x,z) + S^-(q_{z,j})\,v_{z,-}^{II}(q_{z,j},x,z)$$
$$= \sum_j \left[\frac{-q_{z,j}}{k_0 Z_0\rho_z}S^+(q_{z,j})\mathrm{e}^{-iq_{z,j}z} + \frac{q_{z,j}}{k_0 Z_0\rho_z}S^-(q_{z,j})\mathrm{e}^{iq_{z,j}z}\right]G(q_{z,j},x), \qquad \text{(S-12)}$$

where $S^+(q_{z,j})$ and $S^-(q_{z,j})$ are two set of coefficients to be determined. Applying the field continuity condition at $z=0$ and $z=-d$ (the continuity of the pressure field and z-component of the velocity), we are able to completely solve the scattered fields. Firstly, at the metasurface-rigid wall interface($z=-d$), we have

$$v_z^{II}\left(x, z=-d\right)=\sum_j \frac{-q_{z,j}}{k_0 Z_0 \rho_z}\left[S^+\left(q_{z,j}\right)\mathrm{e}^{-iq_{z,j}d}-S^-\left(q_{z,j}\right)\mathrm{e}^{iq_{z,j}d}\right]G\left(q_{z,j}, x\right)=0, \quad \text{(S-13)}$$

then

$$S^-\left(q_{z,j}\right)=S^+\left(q_{z,j}\right)\mathrm{e}^{-2iq_{z,j}d}. \quad \text{(S-14)}$$

Similarly, at the air-metasurface boundary($z=0$), we have

$$\begin{aligned} &\mathrm{e}^{ik_x^{in}x}+\sum_n R_n \mathrm{e}^{ik_x^{r,n}x}=\sum_j\left[S^+\left(q_{z,j}\right)+S^-\left(q_{z,j}\right)\right]G\left(q_{z,j}, x\right) \\ &-\frac{k_z^{in}}{k_0 Z_0}\mathrm{e}^{ik_x^{in}x}+\sum_n \frac{k_z^{r,n}R_n}{k_0 Z_0}\mathrm{e}^{ik_x^{r,n}x}=\sum_j \frac{-q_{z,j}}{k_0 Z_0 \rho_z}\left[S^+\left(q_{z,j}\right)-S^-\left(q_{z,j}\right)\right]G\left(q_{z,j}, x\right) \end{aligned}. \quad \text{(S-15)}$$

Substituting equation (S-14) into equation (S-15), we have

$$\begin{aligned} &\mathrm{e}^{ik_x^{in}x}+\sum_n R_n \mathrm{e}^{ik_x^{r,n}x}=\sum_j S^+\left(q_{z,j}\right)\left(\mathrm{e}^{-2iq_{z,j}d}+1\right)G\left(q_{z,j}, x\right) \\ &-k_z^{in}\mathrm{e}^{ik_x^{in}x}+\sum_n k_z^{r,n}R_n \mathrm{e}^{ik_x^{r,n}x}=\sum_j \frac{q_{z,j}}{\rho_z}S^+\left(q_{z,j}\right)\left(\mathrm{e}^{-2iq_{z,j}d}-1\right)G\left(q_{z,j}, x\right) \end{aligned}. \quad \text{(S-16)}$$

Then we can obtain the coefficient of the *n*-th order diffraction

$$R_n\left(k_x^{r,n}\right)=\sum_j S^+\left(q_{z,j}\right)\left[\frac{k_z^{in}}{k_z^{in}+k_z^{r,n}}I_p\left(q_{z,j}, k_{x,n}^r\right)+\frac{q_{z,j}}{k_z^{in}+k_z^{r,n}}I_v\left(q_{z,j}, k_{x,n}^r\right)\right], \quad \text{(S-17)}$$

where $I_p\left(q_{z,n}, k_{x,n}^r\right)$ and $I_v\left(q_{z,n}, k_{x,n}^r\right)$ refer to the following integrals

$$\begin{aligned} &I_p\left(q_{z,j}, k_{x,n}^r\right)=\frac{1}{L}\sum_m l\left(\mathrm{e}^{-2iq_{z,j}d}+1\right)\int_{m(l-1)}^{ml}G\left(q_{z,j}, x_m\right)\mathrm{e}^{-ik_x^{r,n}x_m}\,dx \\ &I_v\left(q_{z,j}, k_{x,n}^r\right)=\frac{1}{L}\sum_m \frac{l}{\rho\left(x_m\right)}\left(\mathrm{e}^{-2iq_{z,j}d}-1\right)\int_{m(l-1)}^{ml}G\left(q_{z,j}, x_m\right)\mathrm{e}^{-ik_x^{r,n}x_m}\,dx \end{aligned}. \quad \text{(S-18)}$$

Based on equation (S-17), for arbitrary incident wave, we can figure out the Floquet-Bloch mode stimulated inside the metasurface slab, which will contribute to the free space diffraction orders.
